# Brazilian data of renal cell carcinoma in a public university hospital

**DOI:** 10.1590/S1677-5538.IBJU.2014.0452

**Published:** 2016

**Authors:** Pedro Aguiar, Tiago Costa Pádua, Daiane Pereira Guimarães

**Affiliations:** 1 Departamento de Oncologia, Unifesp, São Paulo, Brasil

**Keywords:** Renal carcinoma, nephrectomy, sunitinib, survival

## Abstract

**Purpose:**

Among renal malignancies, renal cell carcinoma (RCC) accounts for 85% of cases. Stage is a relevant prognostic factor; 5-year survival ranges from 81% to 8% according to the stage of disease. The treatment is based on surgery and molecularly targeted therapy has emerged as a choice for metastatic disease.

**Materials and Methods:**

Retrospective study by reviewing the medical records of patients with RCC treated in the last 10 years at UNIFESP. The primary end point of this trial was to evaluate the overall survival (OS) of the patients. The secondary end point was to evaluate the progression-free survival (PFS) after nephrectomy.

**Results:**

118 patients with RCC were included. The mean age was 58.3 years, 61.9% men; nephrectomy was performed in 90.7%, clear cell was the histology in 85.6%, 44 patients were classified as stage IV at diagnosis. Among these, 34 had already distant metastasis. 29 patients were treated with sunitinib. The median OS among all patients was 55.8 months. The median PFS after nephrectomy was 79.1 months. Sarcomatoid differentiation HR29.74 (95% CI, 4.31-205.26), clinical stage IV HR1.94 (95% CI, 1.37-2.75) and nephrectomy HR0.32 (95% CI, 0.15-0.67) were OS prognostic factors. Sunitinib had clinical activity.

**Conclusions:**

Patients treated in our hospital achieved median OS compatible with literature. Nevertheless, this study has shown a high number of patients with advanced disease. For patients with advanced disease, treatment with sunitinib achieved median OS of 28.7 months, consistent with the literature.

## INTRODUCTION

Renal cell carcinomas (RCCs) account for 80–85% of all primary renal malignancies and 2–3% of all cancers in adults (1). Although they are the sixth most common malignance in the USA, there is not any Brazilian epidemiologic data. RCCs are among the most lethal urologic cancers. In the United States, it is estimated that there were 63.920 new cases of kidney and renal pelvis cancer in 2014, and an estimated 13.860 people died of this disease, according to data from the national registry (2).

RCC is more common in male individuals, who outnumber female patients in a ratio of 3:2, and is most frequently diagnosed in the elderly, with a median age of 64 years. There are several established risk factors such as smoking, hypertension, acquired cystic disease of the kidney, and obesity. Most cases are sporadic; however, 2–3% are hereditary. Several genetic syndromes are associated with this disease, of which the best known and most studied is Von-Hippel Lindau disease, which is associated with clear cell carcinoma and other neoplasms (3). RCC is divided into several subtypes, according to histological features, genetic alterations, and cellular origin. Clear cell carcinomas arise from the proximal tubule and are the most common. Other RCC subtypes include papillary, chromophobe, oncocytic, and collecting-duct carcinomas (4). Translocation carcinoma is a specific subtype of RCC that tends to occur in younger patients and is associated with genomic alterations on chromosome Xp11.2, expression of transcription factor E3, and a poor prognosis (5, 6). Some cases of RCC show sarcomatoid differentiation and are related with poor prognosis.

Recently, an increase in incidence has been observed for all stages of RCC; most frequently, these tumors are detected incidentally in asymptomatic individuals. When in early stages, the gold standard of treatment for these tumors is surgery (radical nephrectomy or other renal-sparing approaches). The clinical presentation of RCC is undetermined and sometimes the symptoms arise late. Because of these facts, almost 20% of cases are diagnosed as advanced disease (2), and systemic therapy is indicated. For many years, the standard of care was cytokines. Interferon [IFN] is marked by low response rates (around 5%) and several adverse effects (7); however, it still is a treatment option, especially when vascular endothelial growth factor (VEGF) inhibitor is unavailable. Interleukin 2 was another cytokine largely studied and although it was the only that achieved cure in some patients, it was related to serious and sometimes life-threatening adverse events (8).

A better understanding of the pathways involved in RCC pathogenesis has enabled the identification of some targets for therapeutic intervention. The most studied target is the VEGF pathway, that led to the development and approval of sunitinib and other VEGF inhibitors (sorafenib, pazopanib, bevacizumab, and axitinib) (9-11). Several studies have reported better response rates, overall survival, and disease-free survival with VEGF inhibitors than with IFN (12).

The main objective of this study was to evaluate the outcomes of various RCC treatments at a Brazilian public hospital.

## MATERIALS AND METHODS

### Patients

This study included all patients (aged≥18 years) with histologically confirmed RCC, who were treated at Hospital São Paulo, the University Hospital of Federal University of São Paulo, between January 2004 and May 2014. Any disease stage was allowed. The exclusion criteria included patients whose medical records were inadequate and individuals with other synchronous malignancies.

## STUDY DESIGN

This retrospective study followed a quantitative approach; medical records were assessed to collect baseline epidemiological and clinical data, in addition to the information pertaining to RCC therapy. The study was approved by the institutional ethics committee and was conducted in accordance with the provisions of resolution 466/12 of the Brazilian National Health Council and Good Clinical Practice guidelines.

## TREATMENT PROTOCOLS

The physicians were responsible for decision-making regarding treatment. Molecularly targeted therapy for RCC treatment, specifically sunitinib, has been available in the public health system of the state of São Paulo since 2009. Therefore, aspects related to advanced disease treatment were assessed for these two periods, before and after targeted therapy.

Treatment with IFN involves three subcutaneous infusions per week, with an initial dose of 3 million units (MU) in the first week, 6 MU in the second week, and 9 MU thereafter, if tolerated well. Sunitinib treatment was administered at 50mg/day for 4 weeks, with 2 weeks off treatment. Both protocols could be adjusted in accordance with the adverse effects.

## EFFICACY AND SAFETY

The primary endpoint of the study was overall survival (OS), defined as the time from the diagnosis to death from any cause. The secondary endpoint was progression-free survival (PFS), defined as the time from nephrectomy to the first documentation of objective disease progression or death from any cause. The evaluation for PFS was made according to the investigator’s assessment. Imaging studies were performed at intervals set by the physician. Tumor response was assessed by investigators according to the response evaluation criteria in solid tumors (RECIST version 1.1). Therefore, PFS was assessed for a subset of 84 patients who did not have metastasis at diagnosis. The disease was staged at the time of diagnosis according to the guidelines of the American Joint Committee on Cancer version 7.

### Statistical analysis

The demographic characteristics were evaluated with descriptive statistics. Time-to-event analyses were performed using the Kaplan–Meier method. A stratified log-rank test and the multivariate Cox regression model were used to evaluate the potential influences of the patient’s baseline characteristics, including age, sex, nuclear grade (Fuhrman), and disease stage at diagnosis, on median PFS and median OS (13). A univariate Cox regression model was used to evaluate the effects of nephrectomy, or different treatments, towing to the limited size of these groups of patients with metastasis (n=34 and n=68, respectively). A p-value of <0.05 was considered statistically significant, and was calculated up to two decimal places. Data on patients who were lost to follow-up were censored at the time of the last evaluation. All authors had access to the primary data and take responsibility for the veracity and completeness of the data reported.

## RESULTS

### Patients

Between January 2004 and May 2014, medical records of 124 patients with RCC were assessed, but 6 were excluded from the study (5 patients with incomplete data and 1 with synchronous malignancies in the lungs); thus, 118 patients were included in the study. Thirteen patients were lost to follow-up, and their data were censored at the time of the last evaluation.

Table-1 summarizes the baseline characteristics of the patients. The clinical stage at diagnosis was determined in 116 patients: 35 (30.2%) were stage I, 16 (13.8%) stage II, 21 (18.1%) stage III, and 44 (37.9%) stage IV; 34 (28.8%) had metastasis at diagnosis.

**Table 1:** Baseline clinical and treatments characteristics.

	Mean	Lower	Upper	Median	SD
Age	58.3	22	87	58	13.3
Gender	Female	38.1%			
	Male	61.9%			
Histology	Renal Cell	85.6%	Sarcomatoid	2.5%	
Subgroups	Cromophobe	5.1%	NA	6.8%	
Furhman Grade	1	3.4%	3	29.7%	
	2	46.6%	4	10.2%	
			NA	10.2%	
Clinical Stage	1	29.7%	3	17.8%	
	2	13.6%	4	37.3%	
			NA	1.7%	
Metastasis at Diagnosis	Yes	28.8%			
	No	71.2%			
Metastatic Nephrectomy (n=34)	Yes	73.5%			
	No	26.5%			
Metastatic Treatment (n=68)	Sunitinib	42.6%	Suportive Care	38.2%	
	Interferon	4.4%			
	Surgery	4.4%	Not Avaliable	10.3%	

## TREATMENT

**Figure 1:**
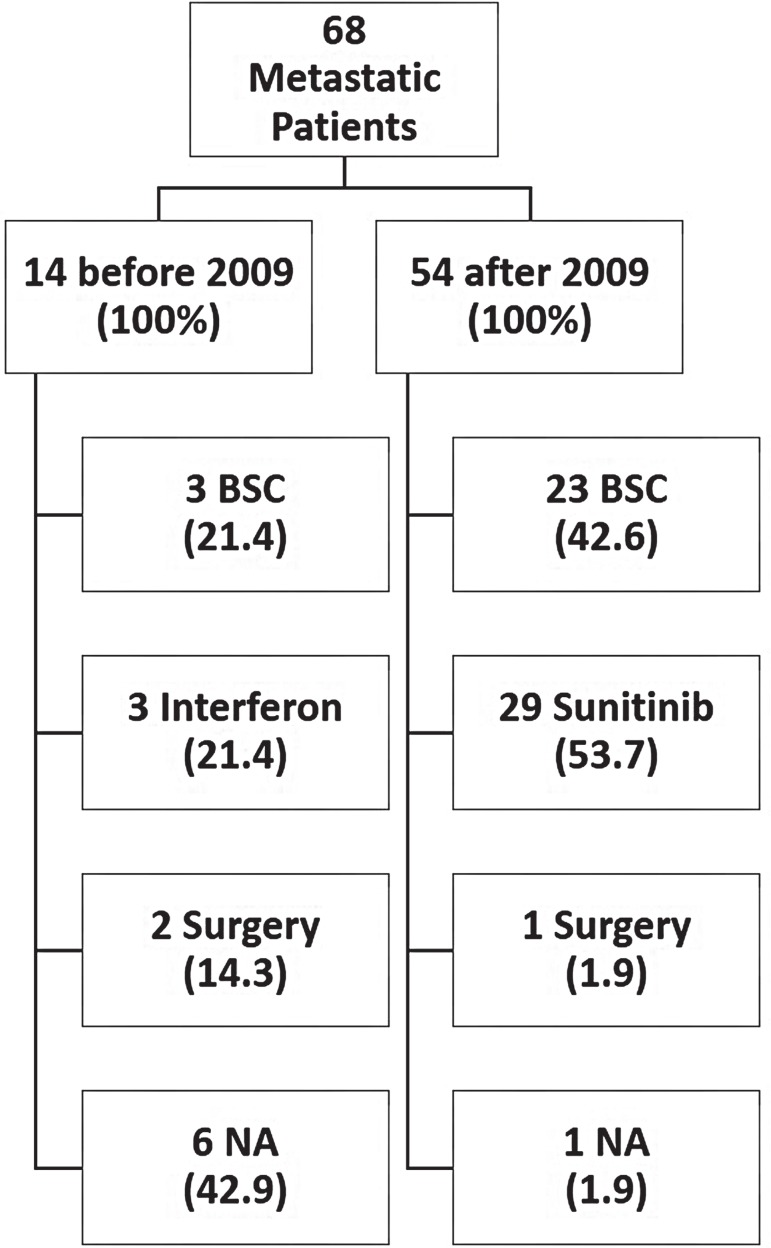
Treatment for metastatic RCC in Hospital São Paulo.

Nephrectomy was performed in 107 (90.7%) cases. Nephrectomy was performed for 25 (73.5%) of the 34 patients who had metastasis at diagnosis. Figure-1 summarizes the treatment options for metastatic RCC; treatment options have been represented as before and after 2009 in order to account for the availability of sunitinib.

**BSC =** Best Supportive Care; **NA =** Not Avaliable.

**Note:** Sunitinib had been available in public health system since 2009.

## PROGRESSION-FREE AND OVERALL SURVIVAL

The median PFS was 79.1 months for all patients who underwent nephrectomy. Nuclear grade IV (Fuhrman) and the clinical stage IV at diagnosis were defined as poor prognostic factors for disease progression, with a hazard ratio (HR) of 2.78 (95% CI, 1.51 to 5.10) and 2.24 (95% CI, 1.49 to 3.37), respectively.

**Figure 2:**
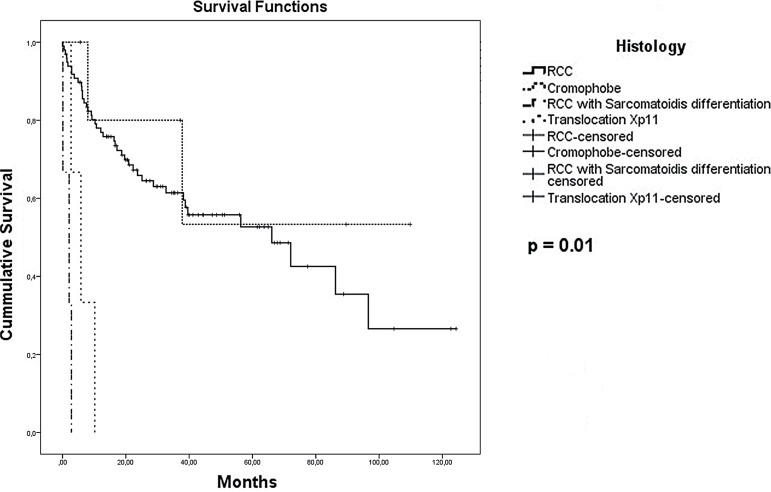
Kaplan-Meier estimates of Overall Survival per RCC histology

**Figure 3:**
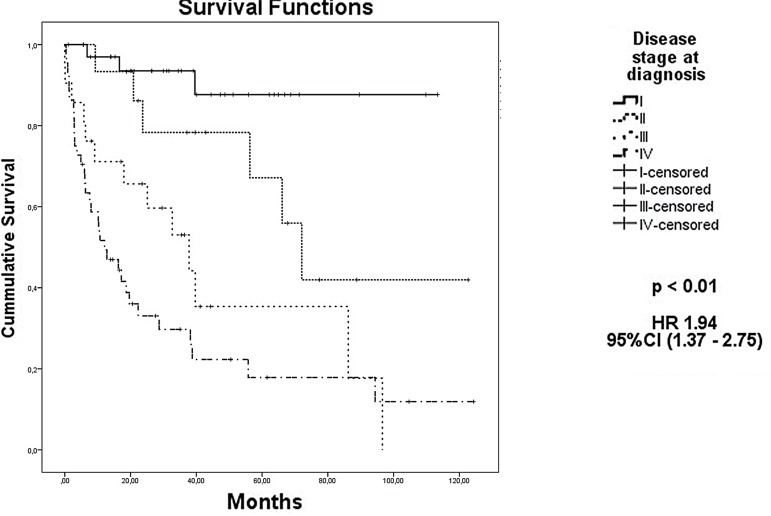
Kaplan-Meier estimates of Overall Survival per clinical stage

**Figure 4:**
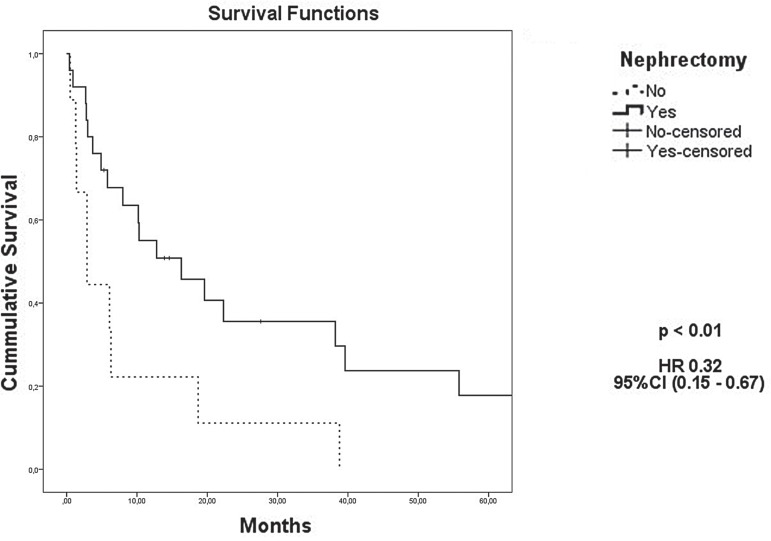
Kaplan-Meier estimates of Overall survival according to nephrectomy for metastatic patients

The median OS for all 118 patients was 55.8 months. The presence of sarcomatoid differentiation and clinical stage IV at diagnosis were defined as poor prognostic factors for death (Figures 2 and 3, respectively), with an HR of 29.74 (95% CI, 4.31 to 205.26) and 1.94 (95% CI, 1.37 to 2.75), respectively. Nephrectomy was defined as a positive prognostic factor (Figure-4), with an HR of 0.32 (95% CI, 0.15 to 0.67), for the 34 patients who had metastasis at diagnosis.

**RCC =** Renal Cell Carcinoma

**HR =** Hazard Ratio; CI95: 95% Confidence Interval

**HR =** Hazard Ratio; CI95: 95% Confidence Interval

**Figure 5:**
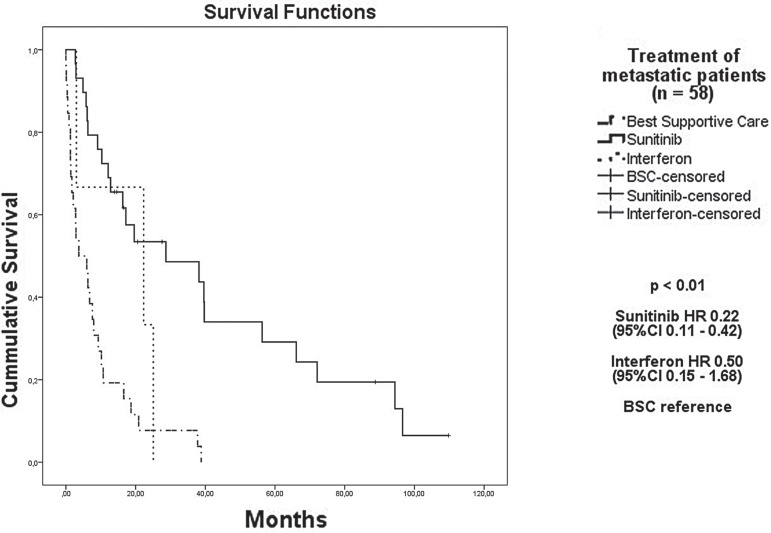
Kaplan-Meier estimates overall survival according to treatment.

**Figure 6:**
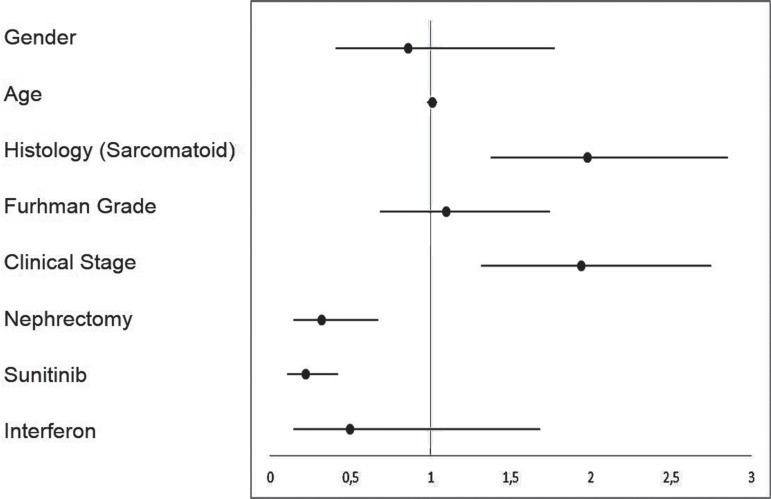
Forest-plot of subgroup analysis for Overall Survival

In addition, treatment with sunitinib was defined as a positive prognostic factor (Figure-5) for the 68 patients with metastatic or progressive disease compared to best supportive care, with an HR of 0.22 (95% CI, 0.11 to 0.42). Figure-6 depicts a forest-plot chart that summarizes the analysis of subgroups.

**BSC =** Best Supportive Care; **HR =** Hazard Ratio; CI95: 95% Confidence Interval

## DISCUSSION

Although RCC is not a rare neoplasm, the Brazilian epidemiology remains uncertain. In addition, there is little information about clinical features of this tumor in our country. Despite of these limitations we believe that the clinical outcomes of RCC in our institution are similar to the international literature. This retrospective study showed that patients treated at Hospital São Paulo had a similar prognosis to that reported in the literature (12). It is not possible to understand the entire Brazilian RCC epidemiology and clinical outcomes only based in these results because of the retrospective design of the trial and the short number of included patients.

The clinical stage at diagnosis was the most important prognostic factor of the disease, as was already expected. In this study, the median OS was not reached for patients with stage I, and it was 72.1 months for stage II, 37.8 months for stage III, and only 12.1 months for stage IV; the five-year survival rate in the aforementioned stages was 85%, 65%, 38%, and 19%, respectively. A majority of patients had advanced disease at diagnosis and it is not possible to correlate this finding with socioeconomic features because in this study some demographic data was not available.

Sarcomatoid differentiation was a poor prognostic factor. Some trials had assessed this issue with similar results. Interestingly, it was also observed in a Mexican trial that has assessed clinical and pathological aspects related to poor prognosis among patients with Stage III or IV RCC (14). In this study, 126 patients were included and 8.7% had sarcomatoid differentiation (14). After a multivariate Cox regression analysis, the risk of cancer-specific death was more than 3 times higher among individuals with sarcomatoid differentiation (14). Lymph node invasion was also a poor prognostic factor; however, our trial did not evaluate this aspect.

Nephrectomy is the most important treatment for RCC, even in the advanced stages. Support for this approach comes from an observational study of 314 patients treated with molecularly targeted agents, including 201 patients who underwent cytoreductive nephrectomy (15). Patients who underwent cytoreductive nephrectomy had a significantly longer OS than those who did not have surgery (19.8 versus 9.4 months, p<0.01) (15). This benefit persisted on multivariate analysis after adjusting for other known risk factors (HR 0.7, 95% CI, 0.5 to 1.0) (15). In our cohort, of the 34 patients who had metastasis at diagnosis, 25 were eligible for nephrectomy; of the remaining, only 3 (33.3%) were treated with molecularly targeted therapy alone. The remaining 6 patients (66.6%) were treated with best supportive care (BSC) because of a poor performance status. We found similar results, the median OS among patients whom underwent nephrectomy was 16.3 versus 2.9 months in the control group. However, this large difference might have been overestimated owing to the small number of patients and the comparison between patients who underwent surgery versus those who received BSC alone. In this study, there were two cases of complete remission of metastasis after nephrectomy, and the patients have remained free of disease for 8.5 and 10 years. Another patient underwent surgical removal of pulmonary metastasis, and has remained free of disease for 5 years. Based on these results, even the resection of metastases should be encouraged when appropriate.

In the recent years, some trials are providing data on the comparison of different molecularly targeted agents. The efficacy of sunitinib was firstly demonstrated in a phase III study of 750 patients with metastatic RCC who had not received prior systemic therapy. Patients were randomly assigned to sunitinib or IFNα treatments. Sunitinib resulted in a higher overall response rate (47% versus 12%, respectively), a longer PFS (median PFS of 11 versus 5 months, HR 0.54), and a longer OS (median OS of 26.4 versus 21.8 months, HR 0.82, 95% CI, 0.67 to 1.00) (12). In the state of São Paulo, sunitinib was available after 2009 for patients receiving treatment under the Brazilian Health System (Sistema Único de Saúde). Our study assessed 54 patients with metastasis after 2009; 53.7% of patients were treated with sunitinib, whereas 42.6% received BSC. A high proportion of patients underwent best supportive care primarily because of the patient’s poor performance status. Sunitinib therapy was effective in improving OS; the median OS with sunitinib was 28.7 months, versus 3.7 months with BSC. In the Group of patients who received treatment before 2009, of 14 patients with metastasis, 3 were treated with IFN. No statistical significant benefit was obtained with the use of IFN compared to BSC (HR 0.50, 95% CI, 0.15 to 1.68). To date, there are few studies describing the efficacy and safety of sunitinib in a Brazilian population. In 2012, Smaletz et al. have shown a cohort of Latin American patients who has achieved long term clinical benefit (more than 20 months) with the use of sunitinib. Only 29 patients were included and it was hypothesized that young patients with good performance status had the highest benefit (16).

There are some limitations in this study, of which the most important are the small number of patients and the retrospective design. Every evaluation must be done carefully. Furthermore, it is impossible to demonstrate the superiority of one treatment over another because of the retrospective design. Many patient’s, especially those with localized disease, were lost to follow-up. Moreover, incomplete and fragmented medical records complicated some analysis. Regardless, this study presents relevant data for subjects treated in a Brazilian public university hospital.
